# Left Ventricular Rupture Post Mitral Valve Replacement

**DOI:** 10.4137/cmc.s2533

**Published:** 2009-09-23

**Authors:** Sameh I. Sersar, Ahmed A. Jamjoom

**Affiliations:** 1 Division of Cardiothoracic Surgery, Department of Cardiovascular Diseases, King Faisal Specialist Hospital and Research Center, Jeddah, Saudi Arabia.; 2 Mansoura University, Cardiothoracic Surgery Department, Mansoura, Egypt.

**Keywords:** left ventricular rupture, mitral valve replacement

## Abstract

Prevention is better than cure best applies here. As per many authors, posterior leaflet chordae preservation prevent Left ventricular rupture (LVR) and preserve LV geometry. We are presenting here 5 types of left ventricular rupture (LVR) post Mitral valve replacement (MVR) with different methods to repair with the advantages and disadvantages of each. The mortality rate is still very high despite the advances in cardiac surgery. Many therapeutic approaches have been adopted. Yet, none is ideal.

## Introduction

Posterior leaflet and chordae preservation prevent LV rupture and preserve LV geometry.^1^ We are presenting here 5 types of left ventricular rupture with different methods to repair and the advantages and disadvantages of each.

## Case Report

**Figure 1.**
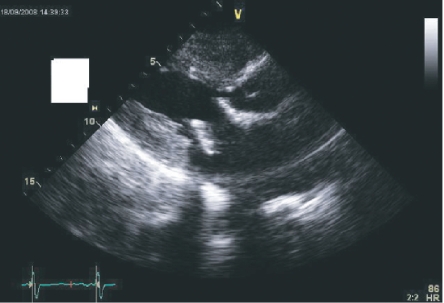
PRE REDO MVR Echo showing significant narrowing of the LVOT and abnormal position of the bioprosthesis.

**Figure 2.**
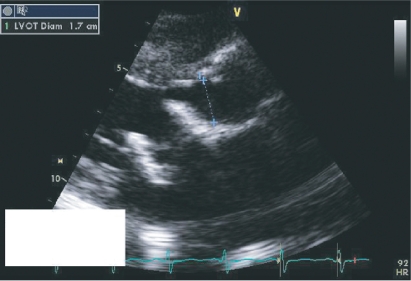
PRE REDO MVR Echo showing significant narrowing of the LVOT and abnormal position of the bioprosthesis.

**Figure 3.**
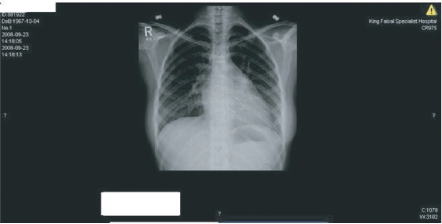
PRE REDO MVR Chest X Ray suggestive of abnormal position of the bioprosthesis.

**Figure 4.**
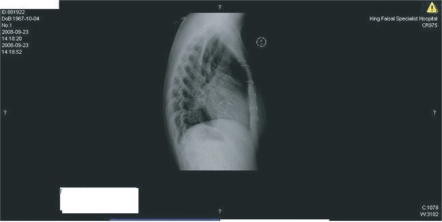
PRE REDO MVR Chest X Ray suggestive of abnormal position of the bioprosthesis.

**Figure 5.**
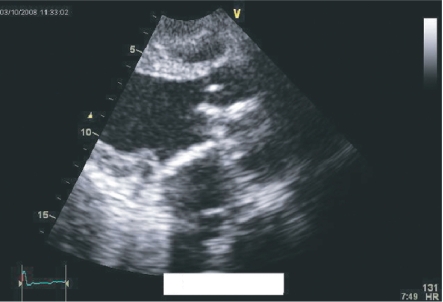
Post-redo MVR echo.

We operated on a 41 year old female with severe mitral bioprosthesis regurgitation 6 years post MVR (bioprosthesis size 31) for rheumatic heart disease. Echo and Chest X ray showed abnormal position of the bio-prosthesis strut, with narrowing of the Left Ventricular Outflow Tract (LVOT); 1.7 cm with a mean systolic pressure gradient of 35 mmhg, (Figs. 1–4). Intraoperatively, it was noted that one of the bioprosthesis struts was abnormally placed and causing pressure and thinning of the posterior wall of the LVOT and both mitral valve leaflets were previously preserved and heavily calcific. We did redo MVR with a mechanical valve size 29. Posterior MV leaflet had to be resected because of severe calcification. Ten hours postoperatively, while preparing the patient for extubation, significant bleeding was noticed and the patient became heamodynamically unstable. Thus, the patient was re-explored. Although there was significant amount of bleeding coming from the area posterior to the aortic valve annulus, it was difficult to identify the bleeding site. Therefore, cardiopulmonary bypass (CPB) was restarted, aortic cross clamp was applied, and cardioplegia was given. Left atrium was opened, there was no problem with the new mechanical mitral valve and no tears were identified. The tear was in the posterior wall of the LVOT just below the left coronary cusp and opposite the intertrigonal area nearer to the left fibrous trigone. We were able to repair the tear only after completely transecting the aorta above the aortic valve. Proper Visualization was impossible without transecting the aorta like mini transplantation. The patient was transferred to intensive care unit in a stable condition and postoperative ECHO was good (Fig. 5). Unfortunately, she died eight days later, due to sepsis and Disseminated Intra-vasular Coagulopathy. Prevention is better than cure best applies here. As per many authors, posterior leaflet and chordae preservation prevent LV rupture and preserve LV geometry.^1^

This may be the first report of a posterior LVOT wall rupture post redo MVR. From our point of view, this new type of LV rupture may be due to the abnormal position of the mitral valve bioprosthesis strut, causing significant pressure and weakness of the posterior wall of the LVOT.^2^ The cause of the mitral valve improper positioning may have been due to the over preservation of the posterior mitral valve leaflet leading to abnormal position of the bioprosthesis. It has been also reported that some of the preservation techniques may cause alterations of the left ventricular geometry causing rupture of the papillary muscles, systemic embolisation, or dehiscence of the mitral leaflets from the malposition. The risk of LVOT Obstruction (LVOTO) in patients with septal hypertrophy undergoing anterior leaflet preservation has been emphasised. Despite these concerns, the current evidence suggests that sub-valvular apparatus preservation (SAP) does result in better outcomes and surgeons should apply relevant techniques that have been described in the literature for eliminating LVOTO obstruction after SAP.^3^,^4^

In addition to the New York University experience to reduce the LV tear during mitral valve surgery which includes avoidance of undue traction on the valve leaflets during removal, careful insertion of sutures into the mitral annulus, avoidance of deeper sutures that penetrate the ventricular muscle beneath the annulus, avoidance of left ventricular vents, avoidance of lifting of the apex of the heart once the prosthetic valve had been inserted and use of translucent obturators constructed so the position of the posterior post of the prosthetic valve could be observed before the prosthesis was inserted.^5^,^6^ We advise not to over preserve the posterior leaflet at the expense of the position or size of the prosthesis and or LVOT. Thorough analysis of the ECHO and chest x ray preoperatively is helpful especially in redo mitral valve surgery.

RUPTURE of the left ventricle post mitral valve replacement (MVR) is an infrequent but highly lethal complication, occurring historically in up to 14% of patients operated upon.^5^,^7^,^8^

LV Rupture post MVR was first reported in 1967 by Roberts and Morrow who described the autopsy findings in two patients. From 1967 to 1985, less than 100 patients have been described but it is almost certain that many other cases have never been reported.^5^

It is now seen less often than 15 to 20 years ago. None the less, the persisting 75% mortality makes welcome any new insight into its prevention and management.^8^,^9^

Mortality varies between 50%–93% with or with cardiopulmonary bypass. External repair is followed by a 67% survival and the internal approach, by a 27% survival.^9^

It has been reported to account for up to 18% of all deaths after MVR.^10^

Miller referred to a 1977 report by Cobbs of six cases of transverse ventricular rupture and suggested that the Type I and Type II classifications suggested by Treasure should be supplemented with an additional type (Type III).^11^,^12^

## Types of Left Ventricular Rupture Post MVR

### According to the site

#### Type I

It is the commonest type. It is located at the atrioventricular groove. This remains the most common site and can be seen in the following circumstances:

- Heavily calcified mitral valve annulus.
- Cases of bacterial endocarditis with mitral valve annular abscess.
- Resection of the posterior leaflet and chordae with placement of subannular sutures for valvular replacement, with consequent local trauma, hematoma, and rupture.
- Improper inspection of the left ventricular posterior wall after MVR by lifting the heart using the atrioventricular groove as a fulcrum.

#### Type II

It occurs at the base of the papillary muscles, primarily due to excessive resection of the posterior papillary muscle, with local hemorrhage and rupture. In type II perforation etiologic agents are: (1) intrinsic myocardial disease of ischemic, rheumatic, ar infectious origin; (2) technical injury to the ventricular wall during excision of the papillary muscles.

#### Type III

It is located between Type I and Type II lesions, and is most often related to posterior left ventricular wall trauma, due to a high profile or large prosthetic valves, often in combination with a small left ventricular cavity. With a bio-prosthetic valve, injuries to the left ventricular wall are known to occur due to penetration of the valve struts in the posterior myocardium. The evolution in techniques of mitral valve surgery over the past two decades has lowered significantly the occurrence of these complications and, in reality, Type II and III have been virtually abolished, primarily due to the following technical reasons:

1. The prevalence of mitral valve repair versus replacement.
2. The preservation of mitral valve leaflets and papillary muscles attachments during mitral valve replacement.
3. The struts of the new bioprosthetic valves have a lower profile and are held in an “inward position” by sutures, lessening the possibilities of trauma to the posterior wall. Recommended technique of lowering the prosthetic device with strut sutures in place is a must and should prevent the penetration of the struts in the posterior myocardium.
4. The abandonment of high-profile prosthetic valves.
5. Awareness of the problem and improved technical guidelines also lessen the incidence of this complication.^13^,^14^

#### Uncommon sites

1. The tear is not in the posterior atrioventricular wall, but in the lateral wall, left side of left anterior descending artery (LAD) opposite the anterolateral commissure of mitral valve to which the chordae tendineae and papillary muscles are adhered.^14^
2. Torn posterior wall of the left ventricle out flow tract opposite the inter-trigonal area nearer to the left fibrous trigone.^2^

#### According to the timing

Early tears occur in the operating room before or after weaning from CBP. Delayed tears present hours to days after leaving the operating room. Late tears appear days to years after MVR and present usually as LV pseudoaneurysms (LVPA).^15^

The early rupture comprises two-third of LV ruptures following MVR. The mortality rate in these patients approaches 50% despite early treatment.^16^

## Risk Factors and Pathogenesis

Preexisting intrinsic myocardial disease especially ischemia, rheumatic and or infectious processes have also been implicated.^14^–^17^ Older age, hemodialysis, and end diastolic diameter less than 50 mm are significant risk factors for LV rupture after MVR. Preservation of the basal chordae of the posterior leaflet is important to prevent LV rupture.^17^

Most of the ruptures are attributed either to technical maneuvers in the operation or to stretch injury produced by the untethering of the left ventricle through removal of the mural leaflet of the mitral valve.^10^–^14^

The “untethered loop” hypothesis proposed by Cobbs in 1980 based on autopsy findings in seven cases, seemed to be the most reasonable explanation. This concept considers the supporting structures of the posterior ventricular wall to form a loop. The outer portion is composed of longitudinal muscle fibers in the ventricular wall, while the inner portion of the loop consists of the papillary muscles with the chordae attached to the annulus of the mural leaflet. Accordingly, division of mural leaflet chordae can seriously weaken the posterior ventricular wall.^5^

Retraction of the left ventricle when the left atrium is fixed by adhesions from a previous operation; extensive resection of a papillary muscle; too large prosthesis; high-profile valves; impingement by a valve strut; presence of deep sutures in the myocardium; mechanical injuries to the left ventricle; forceful retraction; and inadvertent damage to the annulus are blamed. Perforation of the ventricular wall in the region of the papillary muscles may occur during removal of the mitral valve particularly if this is being done under conditions of ischaemic arrest with a flaccid heart.^12^–^14^

The potentially harmful effects of intraoperative hypertension in this context were first pointed out. The incidernce of the use of inotropes immediately before the appearance of bleeding is striking.^18^

The coexistence of aortic valve disease seemed to have a protective effect, possibly because the thickened or hypertrophied ventricular myocardium is resistant to tearing. Associated ischaemic heart disease was not a risk factor as most patients had normal coronary arteriograms. The pathogenesis in type I rupture is worse than type II; However a clear separation into the classification is not always possible.^19^

## Diagnosis

The main clinical presentation is either unstable hemodynamics after CBP weaning, Failure to wean off CBP, Massive bleeding from the LV in the operative field or through the chest tubes, Ventricular arrhythmias and or abrupt hypotension while pseudoaneurysm is the presentation of delayed type.^15^

Huge dissecting hematoma with left ventricular failure can be also a presentation.^18^

Spellberg and O’Reilly reported 2 cases of false ventricular aneurysm post MVR that were thought to be related to the mobilization of the left ventricle from dense pericardial adhesions. After a mitral valve replacement a pansystolic murmur may not represent mitral regurgitation but may be produced by a false ventricular aneurysm. In addition, mitral regurgitation, with or without a pansystolic murmur, may not be a simple paravalvar leak. left ventriculography is recommended when surgery is contemplated, even in the presence of an aortic valve prosthesis, and even though echocardiography indicates mitral regurgitation.^20^

A large sac of pseudoaneurysm or its expansion can compromise the lumen of the circumflex artery and produce a myocardial infarction. Other potential lethal complications of left ventricular pseudoaneurysm (LVPA) include LV failure, thrombus embolization or rupture of aneurysm and death.^17^

LVPA was reported to occur in 0.8% of cases after MVR The causative factors are similar to those of rupture of the left ventricular posterior wall seen early after MVR. The differences between these pathogeneses can be summarized in two points: the timing of the rupture—early or late onset after MVR—and the status of rupture—free or contained. Karlson and colleagues reviewed 125 collected cases of rupture of left ventricular posterior wall after MVR, in which late rupture presenting as an LVPA was seen only in 12 cases (9.9%).^10^,^14^,^21^

Differentiation from paravalvular leak and prosthetic valve dysfunction is necessary. When the size of the LVPA is small, the patient may be asymptomatic. An LVPA may present as a bulge along the left heart border on chest roentgenogram. A dome-shaped or fistula- like extravasation along the posterolateral wall of the left ventricle is seen by left ventriculography. Doppler or color flow-mapping echocardiography demonstrates blood flow across the orifice of the LVPA. Computed tomographic scan and cardiac magnetic resonance with contrast medium are very helpful for better understanding the spatial relationship of the LVPA to the cardiac chambers and the chest wall, particularly for a large LVPA. True aneurysm is usually found at the site of the previously sutured ventricular perforation and the area was plicated using ‘teflon’ reinforced sutures. Sharratt G.P et al, recommend that all patients who have repair of a ventricular perforation, particularly if this was in the atrioventricular groove, should have left ventriculography performed before discharge.^20^ However with MRA and Echo, the need to ventriculography is decreasing.

## Treatment

The difficulties in the repair of a left ventricular rupture after mitral valve replacement are:

1. The ventricular muscle is friable and cannot hold sutures well.
2. Poor exposure and visualization of the anatomy of the rupture site.
3. Technical inaccessibility for placing sutures through and through the ventricular wall, which is usually near the atrioventricular groove and circumflex coronary artery.^8^,^22^
4. Difficulty determining the precise location of the rupture is usually faced.
5. The actual perforation can be distant from the site of obvious haemorrhage.^12^
6. Although small focal perforations have been repaired in a few patients by external methods alone, the external rupture may represent only the “tip of an iceberg,” giving little clue to the extent of the underlying ventricular rupture. With midventricular ruptures, the tear has often been 4 to 5 cm in length.^5^

Several technical considerations have emerged as important factors in the prevention of leftventricular perforation. These are:

1. All posterior Mitral valve chordae should be preserved if at all possible.
2. Avoidance of extensive excision of calcium when it extends through the annulus, particularly in the area of the posterior-medial cornmissure.
3. Careful and accurate sizing of the valve to the body of the ventricle and also to the area of ventricle immediately under-neath the annulus.
4. Accurate and judiciously limited papillary muscle excision.
5. Avoidance of excessive traction on the ventricle when adhesions are still attached to the left atrium and atrioventricular groove.
6. Avoidance of placing a valve strut near the thin attachment of the atrium to the ventricle.^14^

If a tear occurs bypass should be reinstituted if at all possible and repair performed on a decompressed heart. Bypass grafting to distal branches of the circumflex coronary artery and reduction of afterload with the intra-aortic balloon pump may be helpful. To avoid pressure load, the general consensus is that repair should be accomplished with the CPB on a decompressed heart. After load reduction in these critical situations a Intraaortic balloon pump (IABP) will be beneficial to avoid tension on the left ventricle repair and helps the friable, edematous tissue to heal.^22^,^23^

The basic requirements for successful repair of the tear are closure of the full extent of the tear, placement of sutures into healthy myocardium and not into the injured oedematous tissue in the immediate vicinity of the tear, and sparing the left circumflex coronary artery and its major marginal branches. Removal of the prostehesis, accurate identification of the site of tear. Repairs on CBP have a better chance of success.^9^

### Techniques of repair

Several surgical techniques for the repair of left ventricular rupture, including both internal and external approaches, have been described.

### External approach

The external approach is complicated by the fact that the internal disruption site and the external bleeding point are often at different locations; moreover, the circumflex coronary artery is more prone to injury with this approach than with the internal approach. In addition, although the tear may involve the suture line of the previously placed mitral prosthesis, securing the prosthesis from the exterior of the heart is difficult. The epicardial approach includes left thoracotomy, epicardial tissue sealing, Teflon patch and glue. The internal method is considered the safest and most successful approach, even though the previously placed prosthesis has to be explanted during CPB.^22^,^23^

### Enoventricular repair

Endoventricular Repair includes either the standard pledgetted repair technique, posterior leaflet technique, left atrial appendage flap technique, atrialization of the tear, endocardial patch technique, transaortic patch technique, partial translocation and autotransplantation. Internal repair includes direct closure of the tear with multiple horizontal mattress stitches buttressed with felt, patch closure of the tear and covering the tear with a patch sutured on the intact endocardium of the ventricle and the left atrium across the annulus. There should be some tension at the site where the pledgeted mattress sutures are placed with this technique as compared with the patch exclusion technique.^24^–^27^

Surgical repair of the rupture with and without the aid of CPB resulted in 50% and 7% survival, respectively. With the use of CPB, external repair was followed by a 67% survival and the internal approach, by a 27% survival.^9^,^10^

**Figure 6.**
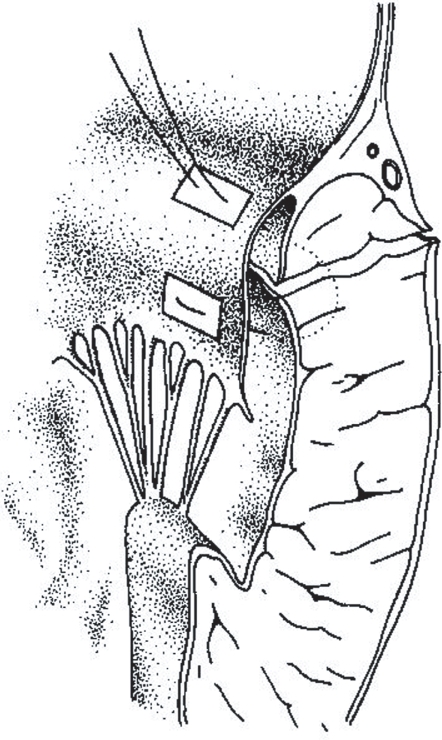
Technique of Repair of Type 1 LV Rupture with the posterior leaflet of the mitral valve. After Izzat MB 1993.

Using the posterior leaflet itself as a buttress on CBP and Cardioplegia helped in; (a) maintaining complete closure of the defect. (b) avoiding any injury to the circumflex artery. (c) ensuring that the longitudinal loop of the left ventricle is left intact^9^ (Fig. 6).

Devineni and McKenzie used the left atrial appendage to cover a type 1 rupture, and sutured it to the left ventricular wall below the defect; on CBP and Cardioplegia.^28^

Treasure and associates placed mattress sutures from the sewing ring of the prosthesis through the ventricle below the tear. The sutures are tied on the epicardial surface over Teflon felt patches on CBP and Cardioplegia.^24^

Azariades and Lennox compressed the area between the left atrium and the base of the papillary muscle using two strips of Teflon and deep mattress sutures passed beneath the coronary vessels in the atrioventricular groove. This technique for repair represents reconstitution of the divided loop between the ventricle and the mitral valve.^29^

**Figure 7.**
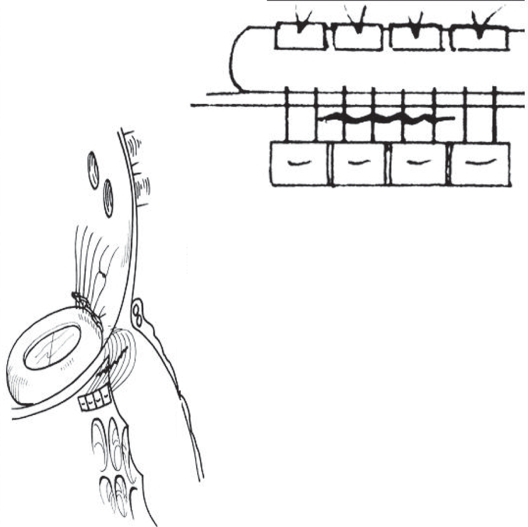
Repair of type 1 left ventricle rupture with pledgeted stitches incorporating the tear and mitral leaflet. After Abid 2002.

Abid et al., 2002 incorporated the tear with the mitral leaflet using pledgetted sutures^23^ (Fig. 7). Autotransplantation; The patient is put back on CPB. The heart is examined and a large hematoma with active bleeding is found in the posterior wall of the left ventricle. The heart is explanted as the standard Stanford technique for heart transplantation: the right atrium, left atrium, and main pulmonary artery are cut at the mid portions, and the aorta is severed between the aortic valve and the aortic clamping site. The explanted heart is placed on a tray, the prosthetic mitral valve is removed and the left ventricle is carefully examined from outer to inner sides. Prolene sutures 3-0 and bovine pericardial patch (Baxter Healthcare Corp, Irvine, Calif) are used to repair the rupture. The bovine pericardium is placed inside the ventricle by transmural stitches in an interrupted manner. Care is taken to avoid injury to the coronary arteries. The prosthetic mitral valve is re-implanted with interrupted sutures. The heart is reimplanted into the mediastinum with the same technique for heart transplantation. After the hemodynamics became stable, CPB is discontinued. Autotransplantation of the heart is an excellent method to rescue patients who develop left ventricular rupture after mitral valve replacement. Cardiac autotransplantation for the treatment of left ventricular rupture was reported by Wei et al and Campanella et al.^8^,^30^

#### Left thoracotomy

**Figure 8.**
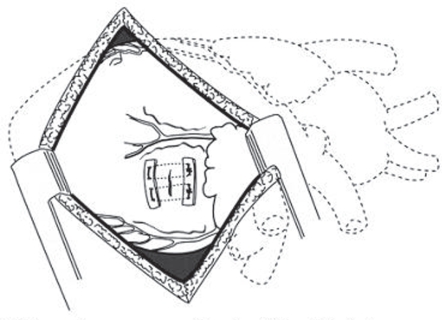
Left thoracotomy exposure and repair of a left ventricular tear. After Victorino 1995.

An intraaortic counterpulsation balloon was placed percutaneously, the sternum closed with chest tube drainage, and the patient repositioned for left thoracotomy. Through a left fifth inter-space thoracotomy and posterior pericardiotomy, the defect is easily visualized and completely repaired with buttressed sutures^31^ (Fig. 8).

#### Repair off-pump with use of a teflon patch and glue

The sternotomy is reopened in the ICU and found an epicardial hematoma and massive bleeding from the posterior wall of the left ventricle. Teflon felt–buttressed interrupted sutures is placed. Histoacryl glue is used to stick a Teflon felt patch (approximately 5 cm in diameter) over the involved area. This emergency procedure is done in the ICU when the clinical condition of the patient is so poor that it is impossible to transfer to the operating theater. The sternotomy is not immediately closed; instead a plastic dressing is sutured over it. The wound is then closed in the ICU 48 hours later.^12^

#### Repair off-pump with epicardial tissue sealing

When rupture is diagnosed and other bleedings sites had been excluded, these patients are left off-pump and protamine was administered. A cell saver was initiated and cell saved blood is washed and re-transfused. Layers of a bio-degradable collagen-system with fibrinogen based coating (Tacho Comb, Nycomed Pharma, Linz, Austria) are carefully placed on the posterolateral epicardium starting from the center of the bleeding site. Manual pressure with a moist gauze for 10–15 minutes on the posterolateral aspect of the beating heart is applied. This procedure was repeated until hemostasis was achieved. It is of utmost importance to spend enough time for the manual pressure procedure of each layer until the collagen system exerted the best gluing effect on the epicardium. In addition, the manual pressure was performed using moisturized gauze that prevented the collagen system from sticking to the surgeon’s glove rather than the epicardium. 3–6 single layers per patient were used to achieve complete hemostasis. With each overlapping layer the epicardial area covered with the collagen fleece increased resulting in decreased bleeding. The total time for the procedure was 45–90 minutes. However, the method is applied off-pump under normo-thermic conditions avoiding the risk of pump-related complications. Once hemostasis is achieved, manual pressure was released and the heart is left in its regular position for an additional 15–20 minutes to ensure sufficient hemostasis. This technique may also be applicable for other large area defects on the heart such as postinfarction ventricular rupture, where ischemic tissues are difficult to suture. This needs to be further evaluated. Carbol et al, strongly recommend this procedure for the treatment of patients with LV rupture or other large area defects on the heart.^32^,^33^

#### Intracardiac patch and extracardiac buttress suture

If the hematoma widely expanded from the posterior to the lateral area of the LV. In order to reduce direct tension to the lacerated ventricular wall, an intracardiac patch (5 × 7 cm, oval shaped) of fresh auto-pericardium is attached with a continuous 3-0 polypropylene suture. The suturing is started from the LV side superior to the remained papillary muscle. Although precise suturing is difficult because of the unclear marginal line between the damaged and normal myocardium, suturing on the healthy myocardium should be attempted. Then, the posterior—lateral atrioventricular wall is covered and secured by the intracardiac patch. Seven interrupted sutures are put through the intracardiac patch, and 9 additional sutures are put on the mitral valve annulas to attach the prosthesis. A mitral prosthesis which is one size smaller than that of the one implanted previously, is selected. Two strips of Teflon were placed on the posterior wall with a buttressed suture in an effort to place the stitches at a sufficient depth while avoiding the circumflex artery. Additionally, in response to continued bleeding, a fibrin sheet, fibrin glue, and GRF glue on the Teflon felt were attached to the repaired region with manual compression. In order to unload the LV afterload, an intra-aortic balloon pump (IABP) is initiated, and then CPB was weaned.^23^,^33^,^34^

#### Use of bioglue surgical adhesive, bovine pericardium, and polytetrafluoroethylene (teflon) patch on cbp and cardioplegia

The BioGlue is applied under and over the Teflon-pledgeted sutures, and an other patch of Teflon felt is glued on top of the Teflon-pledgeted sutures. It is held firmly in place for 2 minutes to achieve good adhesion, and then the aortic cross-clamp is released.^34^,^35^

#### Intracardiac and extracardiac repair

A combination of intracardiac and extracardiac surgical repair techniques used if 2 types of rupture are there. The extracardiac repair involved approximating the edges of myocardium around the tear with large sutures bolstered by strips of Teflon felt, then covering the epicardial hematoma with another porcine pericardial patch, using gelatin resorcinol formaldehyde glue and collagen sheets. The intracardiac repair involved suturing the edges of an oval piece of porcine pericardium to the endocardium around the laceration.^14^,^32^

#### Endoventricular pocket repair using pericardial patch, Teflon felt, and BioGlue

**Figure 9.**
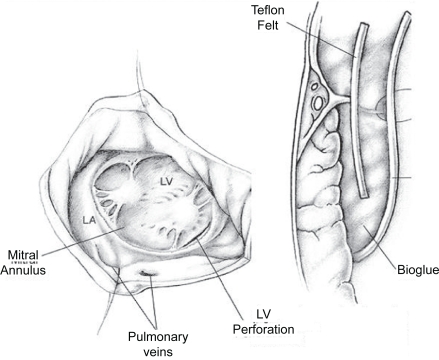
Section through the interventricular groove at the repair site. The valve stiches go through the periocardial pocket. The mitral annulus as seen after excision of the implanted valve showing the location of the tear just beyond the atrioventricular groove. After Masroor S, 2004.

CPB is resumed and the valve prosthesis is excised. A bovine pericardial patch is sewn over this area extending from the ventricular side of the tear out to the atriotomy. A pocket is thus created that is open at the atriotomy. Into this pocket, a piece of Teflon felt saturated in BioGlue is inserted such that the felt lay over the ventricular tear. More BioGlue is poured into the pocket, and gentle pressure was applied to conform the pericardium-felt-BioGlue complex into the shape of the annulus while the BioGlue is congealing. A porcine bioprosthesis is then sutured into place. The open pocket along the atrial suture line is closed with a running 4-0 Prolene suture (Ethicon Inc, Somerville, NJ) along with the atriotomy^35^,^36^ (Fig. 9).

#### Partial translocation

**Figure 10.**
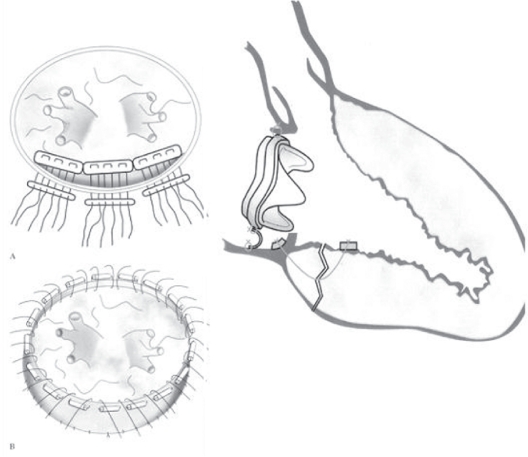
**A**) Nine mattress stitches butressed with Dacron felt were placed from the intact endocardium of the left ventricle through the deep layer of the muscle and pulled through the mitral annulus. **B**) A crescent shaped piece of bovine pericardium was sutured on the left atrial wall above the repaired mitral annulus. Pledgetted mattress sutures for implantation of a prosthesis were placed through the native annulus anteriorly and the newiy constructed annulus posteriorly. Schematic diagram illustrating the partial translocation method for repair of a left ventricular rupture. The tear in the posterior wall of the left ventricle was repaired with mattress sutures buttressed with strips and the posterior mitral annulus was newly constructed with a crescent-shaped piece of bovine pericardium. A Mosaic bioprosthesis was reimplanted at the native annulus anteriorly and newly constructed annulus posteriorly. After Yaku H et al, 2004.

CPB is re-established, and the mitral valve is exposed. After the mitral prosthesis was explanted, the tear involving the endocardium and muscle of the posterior wall is found along the posterior mitral annulus. Mattress stitches buttressed with strips of Dacron (DuPont, Wilmington, DE) felt are put from the intact endocardium of the left ventricle through the deep layer of the muscle and pulled through the mitral annulus. Before tying, gelatinresorcin-formalin (GRF) glue (Cardial SA, Saint-Etienne, France) is put on the tear. A crescent-shaped piece of bovine pericardium (Tissue Guard; Synovis Life Technologies, St. Paul, MN) is then sutured on the left atrial wall above the repaired mitral annulus with 5-0 Prolene continuous sutures (Ethicon Inc, Somerville, NJ), thus creating a new posterior annulus. Pledgetted mattress sutures are placed through the native annulus anteriorly and the newly constructed annulus posteriorly. A Mosaic bioprosthesis is implanted in a paraannular fashion. Considering avoidance of mechanical stress by the prosthesis on the repaired site is crucial to a successful outcome. Constructing a new annulus with bovine pericardium apart from the repaired site and implanting a new prosthesis by using the newly constructed mitral annulus is also crucial^22^ (Fig. 10).

#### Repair by patch and sealing on CPB and cardioplegia

After re-institution of extracorporeal circulation to relieve the strain of the pressure-loaded beating heart, the left atrial incision is reopened. After cross-clamping the aorta, the mitral bio-posthesis is removed to allow careful localization of the site of rupture. Sutures are placed through Teflon patches and the ventricular myocardium at the site of rupture, carefully avoiding the circumflex artery. The mitral bio-prosthesis is re-implanted. This is followed by applying the liquid primer at the site of the ventricular rupture that penetrates into the crevices of the tissue, and then the sealant is applied. Both are exposed to a standard wave length of visible light from a xenon arc lamp, and in 40 seconds, polymerize and change from liquid to a solid gel (photo-polymerization). Then, the sutures placed through the Teflon patches are slightly knotted, avoiding a cut through the friable myocardium. This repair is finished after applying a second layer of the two components of AdvaSeal and the subsequent photopolymerization. The solid gel formed after the light has been applied is highly flexible, elastic, and transparent, and strongly adheres to moist or dry tissue.^15^

#### Intracardiac patch and extracardiac buttress suture

In order to reduce direct tension to the lacerated ventricular wall, an intracardiac patch (5 × 7 cm, oval shaped) of fresh auto-pericardium is attached with a continuous 3-0 polypropylene suture. The suturing is started from the LV side superior to the remained papillary muscle. Although precise suturing is difficult because of the unclear marginal line between the damaged and normal myocardium, suturing on the healthy myocardium should be attempted. Then, the posterior—lateral atrioventricular wall is covered and secured by the intracardiac patch. Interrupted sutures are put through the intracardiac patch, and additional sutures are put on the mitral valve annulas to attach the prosthesis. A mitral prosthesis which is one size smaller than that of the one implanted previously, is selected. Two strips of Teflon are placed on the posterior wall with a buttressed suture in an effort to place the stitches at a sufficient depth while avoiding the circumflex artery. Additionally, in response to continued bleeding, a fibrin sheet, fibrin glue, and GRF glue on the Teflon felt are attached to the repaired region with manual compression. In order to unload the LV afterload, an intra-aortic balloon pump (IABP) is initiated, and then CPB was weaned.^23^,^24^,^36^–^38^

#### Intraventricular patch repair through an extended aortotomy

**Figure 11.**
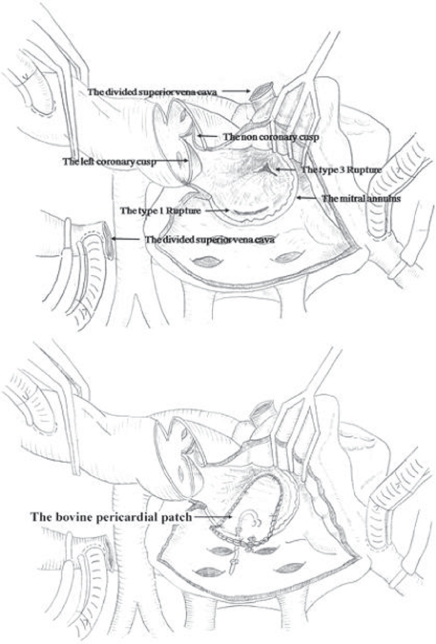
Drawing of the surgeon’s view shows exposure of the mid ventricle through the commissure between the left and noncoronary cusps after the prosthetic valve was removed. The bovine pericardial patch that covered the ventricular ruptures was sutured to the wall of the left atrium. Interrupted, pledgeted mattress sutures were used to reinforce around the continuous suture. After Park CK, et al. 2008.

If the operative surgical field is too deep to be approached through a standard left atriotomy. Approaching the midventricular rupture through an extended aortotomy and transection of the superior vena cava is a good option. The aortotomy incision is extended to the aortic valve annulus between the NCC and the LCC of the aortic valve, similar to the Manoughuian incision. This incision is extended to the standard left atriotomy. Then an intraventricular patch repair, with a large single bovine pericardium covering the first type 1 and the second type 3 left ventricular ruptures, is simultaneously performed through an extended aortotomy. For reinforcement, interrupted, pledgeted mattress sutures are placed around the bovine pericardium. A second repeat MVR with a mechanical prosthetic valve is performed. The aortic annulus and intervalvular aortomitral fibrous continuity is approximated with Prolene 6-0 continuous running sutures at the commissure between the NCC and the LCC. In addition, another plication stitch with 4-0 pledget-buttressed horizontal mattress sutures are placed at the commissure between the NCC and the LCC for good coaptation^38^ (Fig. 11).

### Surgical repair of LVPA

**Figure 12.**
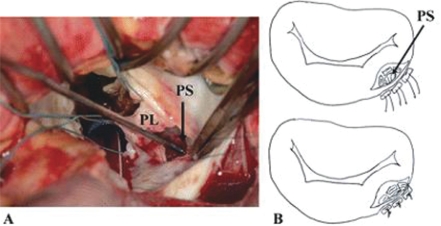
**A**) A 5-mm diameter perforation site existed just below the posterior mitral annulus. (PL = posterior mitral leaflet; PS = perforation site.) **B**) The perforation site was closed directly with three pairs of 3-0 polypropylene sutures and auto-pericardium. After Miura T et al, 2008.

Repair is performed either from inside of the left atrium or from the epicardial surface without opening the cardiac chambers. An internal repair has advantages:

(1) better exposure of the sub-annular apparatus is obtained to make the repair straightforward. (2) additional cardiac abnormalities can be repaired simultaneously. (3) the left circumflex coronary artery is better protected than with an external repair. Its disadvantage is that the mitral prosthesis may need to be explanted for exposure and repair of a rupture site in most cases even though the prosthesis is functioning normally. Also, longer myocardial ischemic time may be required than with an external repair. If there is neither some other intra-cardiac disease nor prosthetic mitral valve dysfunction, an external repair may be chosen. However, extensive adhesion lysis is usually required for this type of repair when approached by a repeated median sternotomy. A left thoracotomy may provide better access to an LVPA in selected cases. The risks entailed by repeat median sternotomy can be avoided. Minimal adhesiolysis is required, and the duration of CPB can be shortened. Presentation of the lesion by retracting the heart is avoided by the left thoracotomy approach. The epicardial tissue around a tear is considered thick and strong enough to hold the sutures late after MVR. Careful sutures on the posterior left atrioventricular groove can avoid damage to the left circumflex coronary artery or the coronary sinus. After securing all the monitoring lines the patient is operated under general anesthesia and femoro-femoral bypass and multi-dose warm blood potassium cardioplegia. Adhesiolysis is performed via para-sternal left atriotomy, interior of the left atrium and left ventricle is inspected. On exploration, a probe entering the pseudo-aneurysm sac located posterior to the left atrium via a tortuous track. The tear is closed using a woven Dacron fabric patch sutured with a 3-0 polypropylene continuous suture. The valve prosthesis is re-implanted using pledgeted 2-0 polyester mattress sutures and free disc movements were confirmed. Left atriotomy is closed with a 3-0 polypropylene continuous suture^19^,^20^,^39^ (Fig. 12). We suggested an algorithm for the different lines of treatment of LVR Post MVR.

## Conclusion

Rupture of the left ventricle post mitral valve replacement (MVR) is an infrequent but highly lethal complication. Early diagnosis, resumption of CPB, proper exposure and complete repair of the tear is necessary to have a better outcome. Endoventricular repair is felt to be better than epicardial approach. we advise not to over preserve the posterior leaflet at the expense of the position or size of the prosthesis and or LVOT. Thorough analysis of the ECHO and chest x ray preoperatively is helpful especially in redo mitral valve surgery.

**Figure 13.**
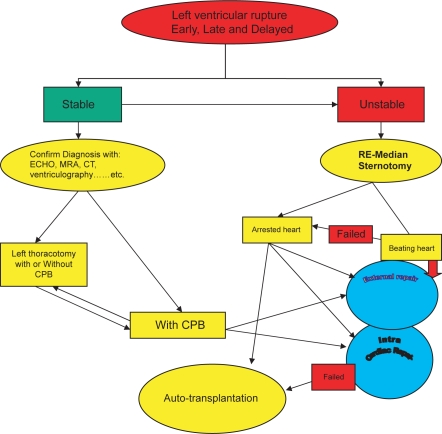
Algorithm suggested for LV Rupture treatment.

We suggested an algorithm for LV rupture treatment (Fig. 13).
